# Neuroprotective Role of DING protein in Fetal Alcohol Spectrum Disorders and Depression

**DOI:** 10.26502/ogr0192

**Published:** 2025-09-26

**Authors:** Nune Darbinian, Monica Hampe, Nana Merabova, Tamara Tatevosian-Geller, Laura Goetzl, Mary F Morrison, Gabriel Tatevosian, Malgorzata Simm, Eric Chabriere, Huaqing Zhao, Shohreh Amini, Michael E. Selzer

**Affiliations:** 1 Center for Neural Development and Repair, Department of Neural Sciences, Lewis Katz School of Medicine at Temple University, Philadelphia, PA 19140, USA; 2 Department of Obstetrics and Gynecology, Gundersen Health System, La Crosse, WI 54601, USA; 3 Department of Obstetrics, Gynecology and Reproductive Sciences, McGovern Medical School at The University of Texas Health Science Center at Houston (UTHealth), Houston, TX 77030, USA; 4 Department of Psychiatry, Lewis Katz School of Medicine at Temple University, Philadelphia, PA 19140, USA; 5 Co-Klinik, Yerevan, Armenia; 6 Department of Biomedical Sciences, Kentucky College of Osteopathic Medicine, 147 Sycamore Street, Pikeville, KY- 41501, USA; 7 Institute Universitaire de France, Aix-Marseille Université, Faculté de Médecine, 13385 Marseille Cedex 5 France.; 8 Center for Biostatistics and Epidemiology, Department of Biomedical Education and Data Science.; 9 Department of Biology, College of Science and Technology, Temple University, Philadelphia, PA 19122, USA.

**Keywords:** fetal alcohol spectrum disorders, depression, DING protein, brain development, gestational age, exosomes

## Abstract

**Introduction:**

An estimated 20% of women consume alcohol during pregnancy, and 10% of women receive antidepressants during their pregnancy. Women with depression are more likely to use alcohol (EtOH) in early pregnancy and are more likely to have a child with one of the fetal alcohol spectrum disorders (FASD). Previously, we provided evidence in rat neurons in vitro that DING phosphatase (p38SJ, a member of the DINGGG family of proteins that has neuroprotective effects under conditions of cellular stress) was neuroprotective against EtOH-mediated toxicity. Now, we examine the effects of DING, alone or in combination with EtOH and serotonergic (5-HT) pathway molecules and drugs, on EtOH-mediated neurotoxicity in human fetal tissues in vitro.

**Methods:**

Human fetal brain tissue was collected between 9 and 23 weeks’ gestation. Primary neurons and neurospheres were prepared from a 16-week fetal brain. Exposures to EtOH and SSRI were assessed by detailed questionnaires. Neurotoxicity/apoptosis was assessed in synaptosome extracts with the Caspase-Glo 3/7 assay and a neurotoxicity microarray. Developmental expressions of DING, serotonin transporter (SERT), and 5-HT1A receptor (5HT1A) protein levels were quantified in the brain, placenta, synaptosomes, serum, and “fetal brain-derived exosomes” (FB-Es) by quantitative western blotting and digital droplet PCR.

**Results:**

Increasing the levels of activated DING, either by addition to culture media or by intracellular overexpression, was associated with increased cell survival. Developmental expressions of DING, in the presence of EtOH- or SSRI-exposure, were investigated in brain and placenta tissues. While 5HT1A, 5-hydroxytryptamine serotonin receptor 1A (5-HT1A) was strongly inhibited under EtOH stress conditions (>10-fold), DING was not only able to reverse the negative effects of stress, as measured by the synaptic plasticity array, but also upregulated the 5-HT receptor (5-fold compared to untreated control) upon treatment of neurons.

**Conclusions:**

Maternal EtOH use is associated with decreased 5-HT receptor and DING protein expression in the fetal brain and with increased neuronal apoptosis. Combined exposure to EtOH and SSRIs may have greater toxic effects than either one alone. DING reversed the toxic effects of EtOH on serotonin receptors in neurons and neurospheres. These findings provide potential mechanisms for the neuroprotective effects of DING, which might suggest neuroprotective approaches to preventing fetal alcohol spectrum disorders (FASD).

## 1. Introduction

EtOH use is generally associated with an increased incidence of depression, and this is also true during pregnancy [1,2,3]. Moreover, prenatal alcohol exposure was linked to mental health problems postnatally [4,5]. Recently, it was demonstrated that several biomarkers of affective dysregulation, including the levels of the serotonin transporter protein (SERT) and brain-derived neurotrophic factor (BDNF) in maternal blood fractions enriched in exosomes originating from fetal brain (“fetal brain-derived exosomes”; FB-E) isolated from maternal blood, were associated with *in utero* exposure to EtOH. Of interest, maternal use of EtOH and SSRI during pregnancy each was associated with changes in fetal brain monoamine pathways, consistent with potential mechanisms for the affective dysregulation commonly seen in fetal alcohol spectrum disorders (FASD) [6]. Although the mechanisms of neurotoxicity brought about by these exposures are incompletely understood, it is still possible that some neuroprotective therapies can be devised to minimize the adverse behavioral outcomes in children. For this reason, we have explored the neuroprotective effects of a novel phosphatase, DING, previously isolated from an injury-induced callus of St. John’s wart [7], which has been used in many cultures as a medicinal plant [8].

### DING protein protects neuronal cells from alcohol-induced injury.

EtOH induces neuronal cell injury and death by dysregulating several signaling events that are controlled, in part, by activation of MAPK/ERK1/2 and/or inactivation of its corresponding phosphatase, PP1. We previously purified a novel 38 kDa protein containing a highly conserved N-terminus motif Asp-Ile-Asn-Gly-Gly-Gly (DING) that has phosphate binding activity (p38SJ/DING), from a callus culture of *Hypericum perforatum* (Saint John’s wort) and from human brain cells [9]. The DING family, named after the conserved N-terminal DING amino acid sequence, encompasses proteins that are implicated in the regulation of many functions in both eukaryotes and prokaryotes, e.g., genome expression and inflammatory responses to HIV [10,11], and neuroprotection from EtOH-induced neuronal injury [9]. Antibodies have been made to this protein and its homologue in the human brain (refs). Although homologous proteins have been found in all organism groups, both prokaryotic and eukaryotic [12], it is not yet known whether these proteins represent true variants or only minor mutations and cleavage products from only one highly conserved gene [13,14,15,16], with functions varying depending on the cell type and environment. The protein recovered from human brain is almost identical to that recovered from St. John’s wort and is labeled by the same antibodies [17]. Therefore, in the present study, we continue to refer to the protein simply as “DING” and its gene as “*ding*”. Treating rat neuronal cells in primary culture with DING protected them against injury induced by exposure to EtOH *in vitro* and diminished their levels of pro-apoptotic proteins, including Bax and activated caspase-3 [9]. These observations may provide novel biological tools for early diagnosis and therapeutic intervention aimed at preventing the neuronal cell death associated with FASD, and possibly for the treatment of other neurological disorders associated with alcohol abuse.

### Novel non-invasive biomarker identifying strategy using fetal brain-derived exosomes.

To evaluate the effects of EtOH on fetal development and to reveal novel early biomarkers for FASD, we have developed techniques to isolate exosomes, presumably derived from fetal brain because they could be immuno-centrifuged using antibodies specific to fetal brain tissues. These “fetal brain-derived exosomes” (FB-E) can cross the placenta and enter the maternal blood, from which they can be isolated non-invasively [18]. We previously studied the expression and activity of neuronal, oligodendrocyte (OL), and synaptic markers, and miRNAs in these FB-E during pregnancy [18,19]. In the present study, we focused primarily on defining the EtOH exposure-associated changes in the expression of DING protein in FB-E and its protective effects on toxicity associated with EtOH and 5HT-related molecules, individually and in combinations.

## 2. Results

Previously, we proposed several biomarkers for FASD in human FB-Es. We also revealed neuroprotective features of exogenous DING *in vitro* against EtOH- and HIV-associated cell toxicity. In the present study, we have used human FB-Es isolated from the blood of pregnant women (Table 1), as well as fetal brain tissues to perform molecular assays, immunocytochemistry, and immunohistochemistry (Table 2), to demonstrate the presence of DING in human FB-Es and other tissues, and to study its neuroprotective features agains EtOH-induced neurotoxicity.

### 2.1. DING is found in human fetal neurons.

**Figure 1:**
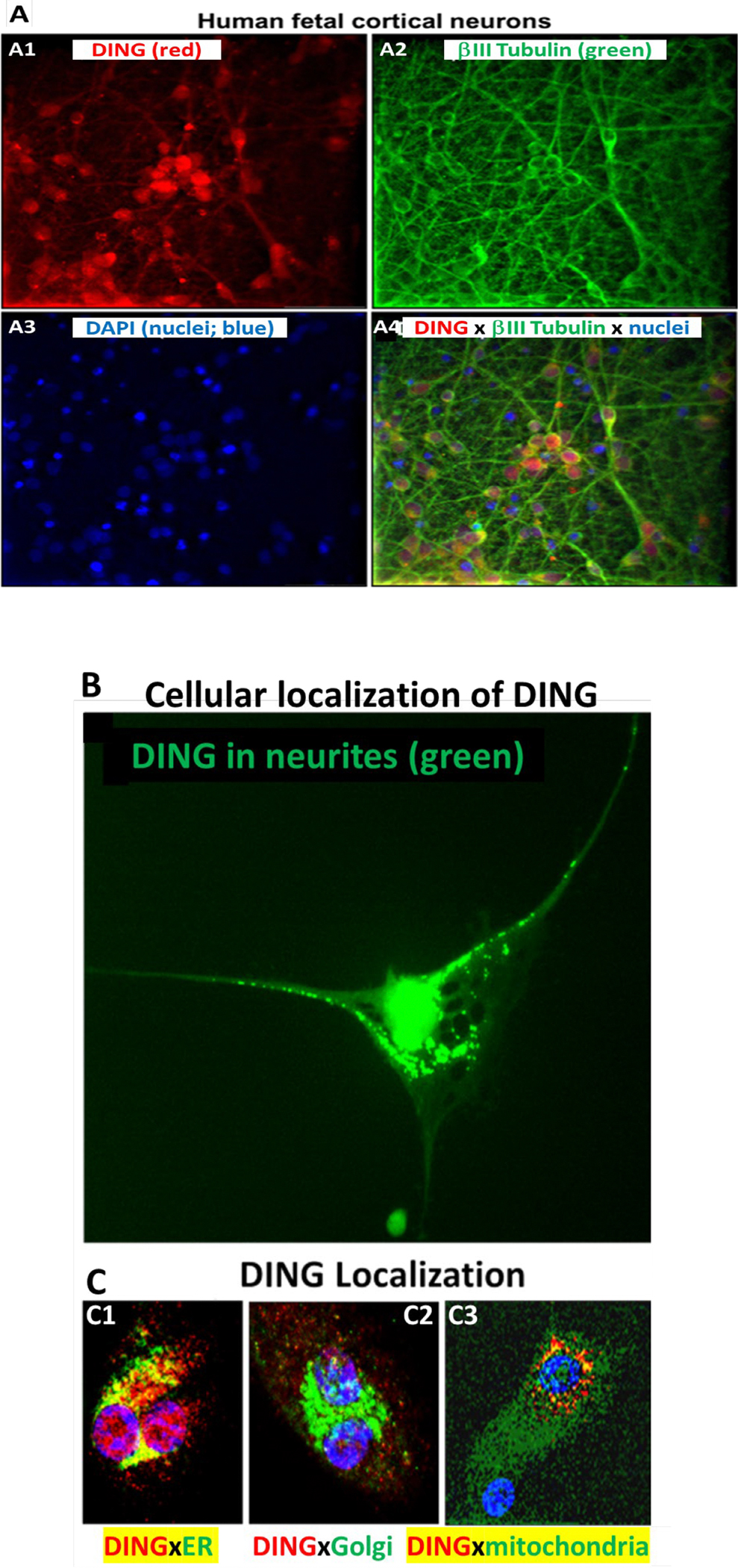
Localization of endogenous DING in human fetal neurons in primary culture. **A.** Immunostaining of human fetal primary cortical neurons for DING (red), βIII tubulin (green), and DAPI (nuclei; blue). **A1:** Human cortical neurons in vitro contain DING (red). **A2:** the neuron-specific marker beta-III tubulin (green). **A3:** the nuclear stain DAPI (blue). **A4:** overlay of A1, A2, and A3. **B.** Cellular localization of DING in human cells. High magnification (40x) image of a human fetal cortical neuron labeled for DING (green). Note the dense label in and around the nucleus and the punctate labeling extending into the neurites. DING is present in neurites (green). **C.** Colocalization with organelles. In this panel, DING is represented in red, and the organelles are green. Colocalization is represented in yellow/orange. **C1**, DING co-localized with ER. **C2**, DING did not colocalize with Golgi. **C3**, DING colocalized with mitochondria.

To confirm the presence of DING in neuronal cells, we performed cytostaining assays using anti-DING protein. DING was localized in neurites and cytoplasm, but mostly in nuclear membranes and nuclei (Figure 1A, 1B), in ER and mitochondria, but not in Golgi (Figure 1C). Thus, DING is expressed in the human fetal brain and present in fetal neurons.

### 2.2. DING protects human neuronal cells from ethanol-induced cell injury.

**Figure 2:**
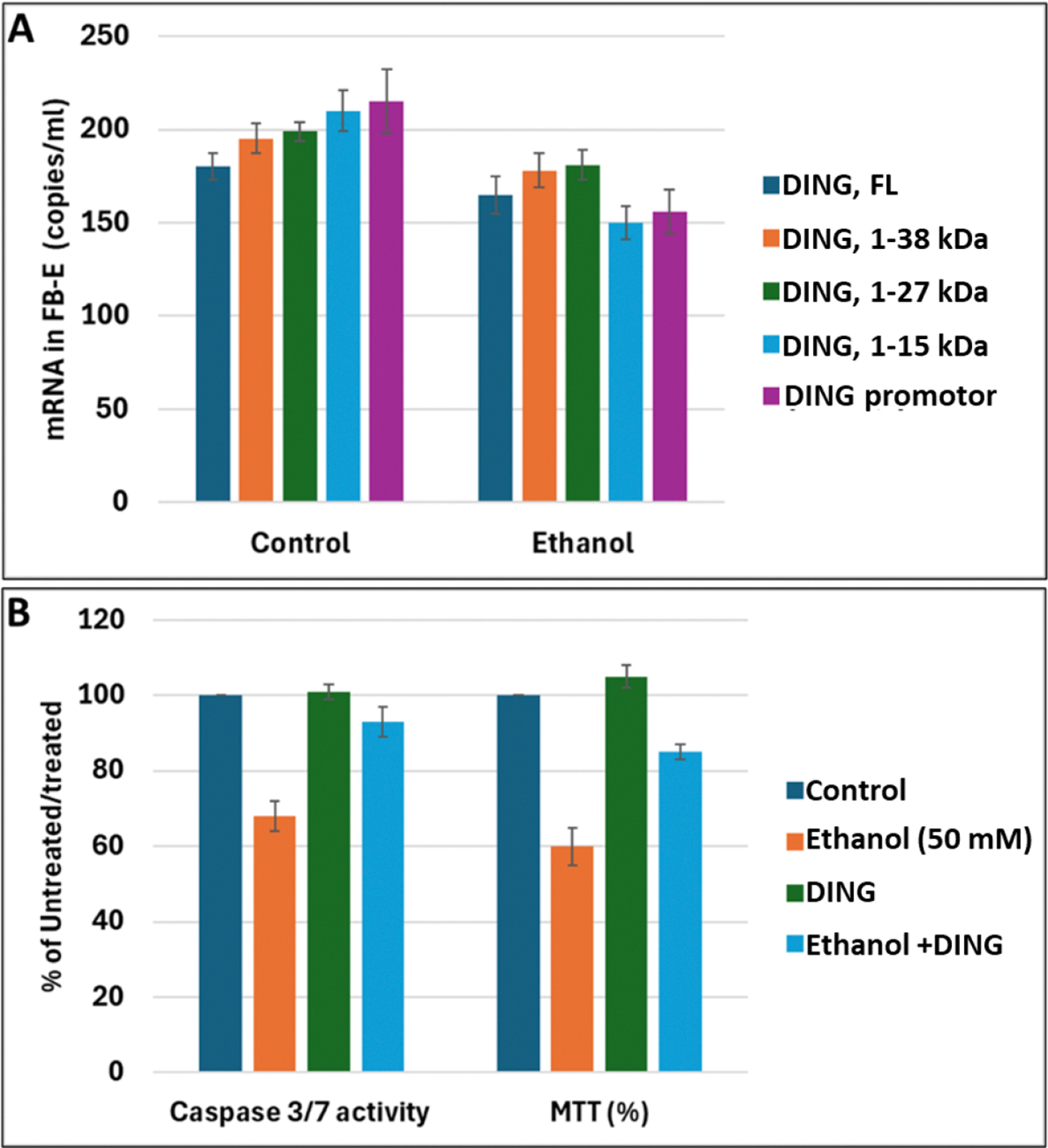
DING protects human neuronal cells from EtOH-induced cell injury. **A.** The mRNA for DING is present in human FB-Es, assayed by ddPCR, using primers for the ding gene and ding promoter spanning a 400 bp region from the N-terminal part of the human ding gene (GeneBank). **B.** DING prevents EtOH-induced activation of neuronal apoptosis. Apoptotic Caspase-3/7 and cell metabolism/activity, MTT, in human cortical neurons treated with EtOH (50 mM) in the presence or absence of DING (300 ng). Cell viability was determined by the methylthiazolyltetrazolium (MTT) assay was quantified by spectrophotometry at 570 nm with a reference wavelength of 650 nm. An equal number of cells was plated in duplicate and then incubated with EtOH. MTT assay illustrating neuronal cell metabolism and activity in the presence of EtOH and DING. The numbers represent the average of three independent experiments. Bar 1 represents untreated cells set as 100%.

To confirm the presence and levels of DING expression in FB-Es, we performed digital droplet PCR (ddPCR). *ding* mRNA (full-length, or its shorter forms, as well as the *ding* promoter region) was expressed in FB-Es from EtOH-exposed fetuses (n=20) and compared with those from unexposed controls (n=20). All *ding* forms were downregulated in EtOH-exposed FB-Es by at least 20–30% (Figure 2A). Exogenously added DING protein increased cell survival in EtOH-treated cells (Figure 2B). Thus, DING is present in FB-Es, fetal brain, blood, synaptosomes, neurons, and neurospheres, and protects human neuronal cells from EtOH-induced cell injury.

### 2.3. DING isoforms suggest that they are cleavage products of a 220kD precursor: EtOH-associated downregulation of DING in human placenta and blood, but not in the fetal brain.

**Figure 3:**
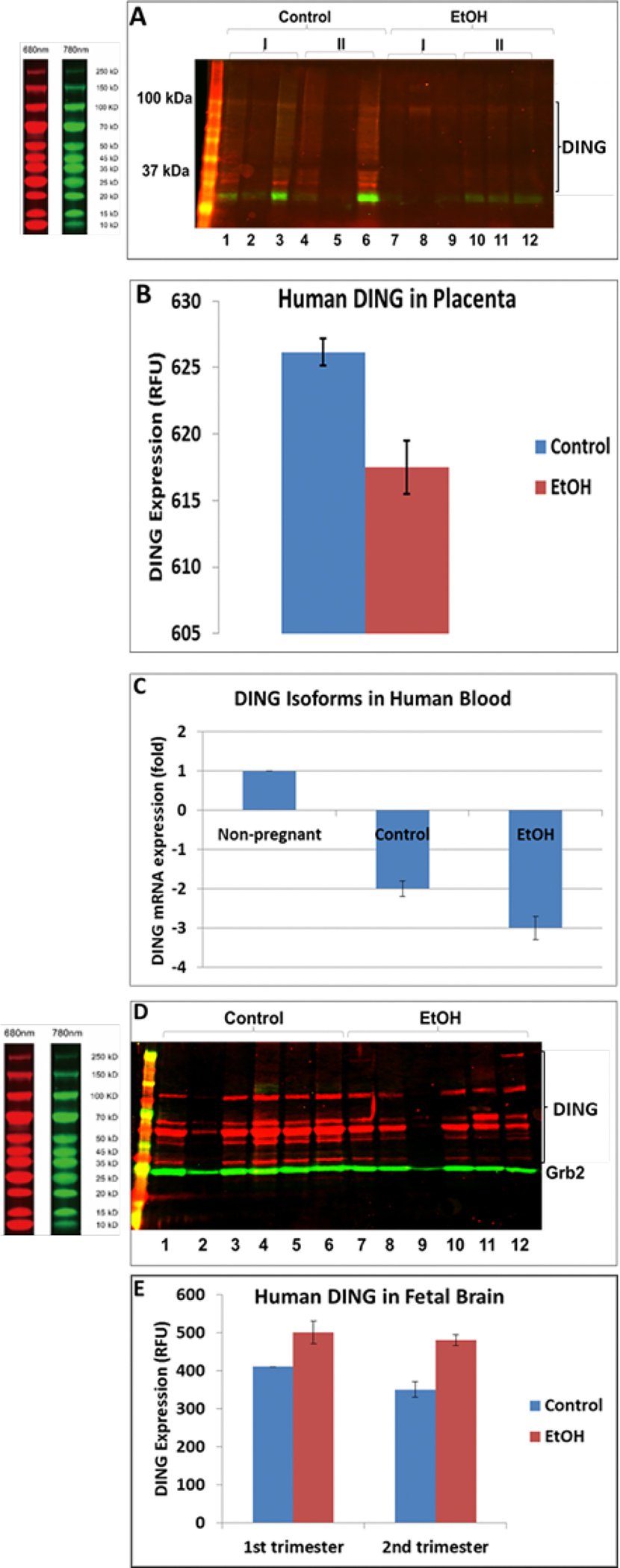
EtOH-associated downregulation of DING protein in human placenta and blood, but not in the brain. **A.** DING protein levels change in EtOH-exposed placentas. Each lane in the western blot represents the contents of a different placenta, i.e., 3 placentas in the 1^st^ trimester, 3 in the 2^nd^ for the control and EtOH-exposed groups. Each well was loaded with 30μg total protein in buffer, adjusted to a total volume of 50μl. Notice the variability in band densities even of Grb2, which is often used as a housekeeping protein for loading controls. In addition to full-length DING downregulation, DING cleavage products in the placenta are also suppressed by prenatal alcohol exposure. **B.** Densitometric representation of pDING levels in human placenta, in relative fluorescence units (RFU). **C.** Downregulation of ding mRNA in maternal blood samples by prenatal exposure to EtOH in the combined 1st and 2nd trimester samples (n=20), detected by qRT-PCR. **D.** Levels of DING protein expression in the human fetal brain are affected by prenatal EtOH exposure. Brain tissues from the 1^st^ and 2^nd^ trimesters were studied. Brain lysates were assayed by a qWestern-blot assay. DING isoforms were differentially changed by EtOH exposure in brain samples. There were several isoforms expressed in the fetal brain, including high and low molecular isoforms and a precursor protein (220 kDa). Decreased levels of HMW and LMW isoforms are seen with EtOH exposure, while the level of the DING precursor was increased. **E.** Densitometric representation of pDING levels in fetal brain, in RFU (relative fluorescence units).

DING levels were decreased in EtOH-exposed placentas. Full-length DING and DING cleavage products were downregulated in alcohol-exposed 1st and 2nd-trimester placentas (Figure 3A–B) and maternal plasma (Figure 3C). At the same time, DING expression was increased in human fetal brains from the first and second trimesters (Figure 3D–E). The fetal brain also expressed several high and low-molecular-weight isoforms and a precursor protein (220 kDa). Decreased high and low MW isoforms are seen with EtOH exposure, while the level of the precursor DING (220 kDa) was increased. This suggests that EtOH may act to inhibit the cleavage of DING precursor protein into active neuroprotective isoforms.

### 2.4. Exogenous DING protects human fetal neuronal cells from EtOH-induced cell injury *in vitro*.

**Figure 4:**
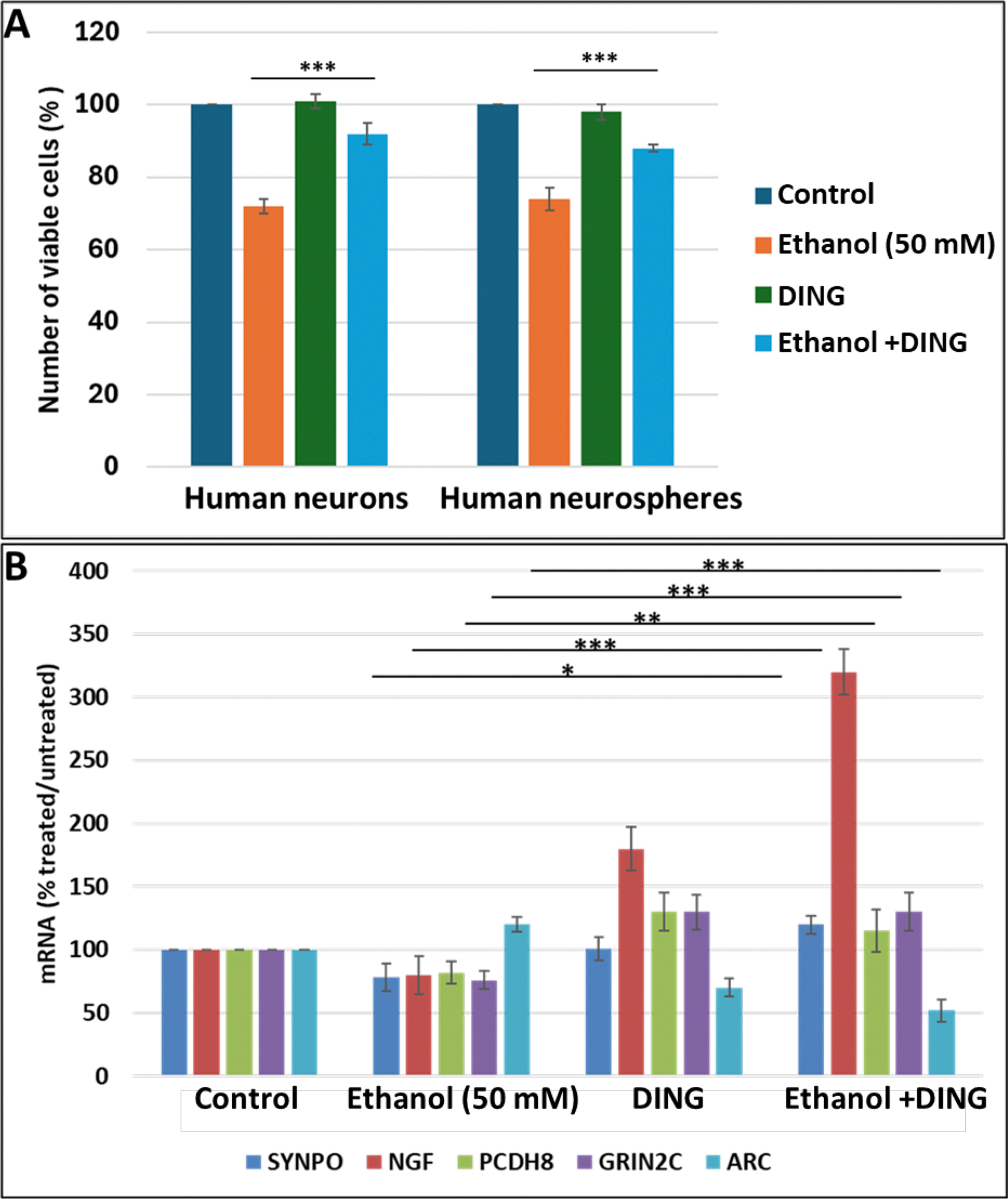

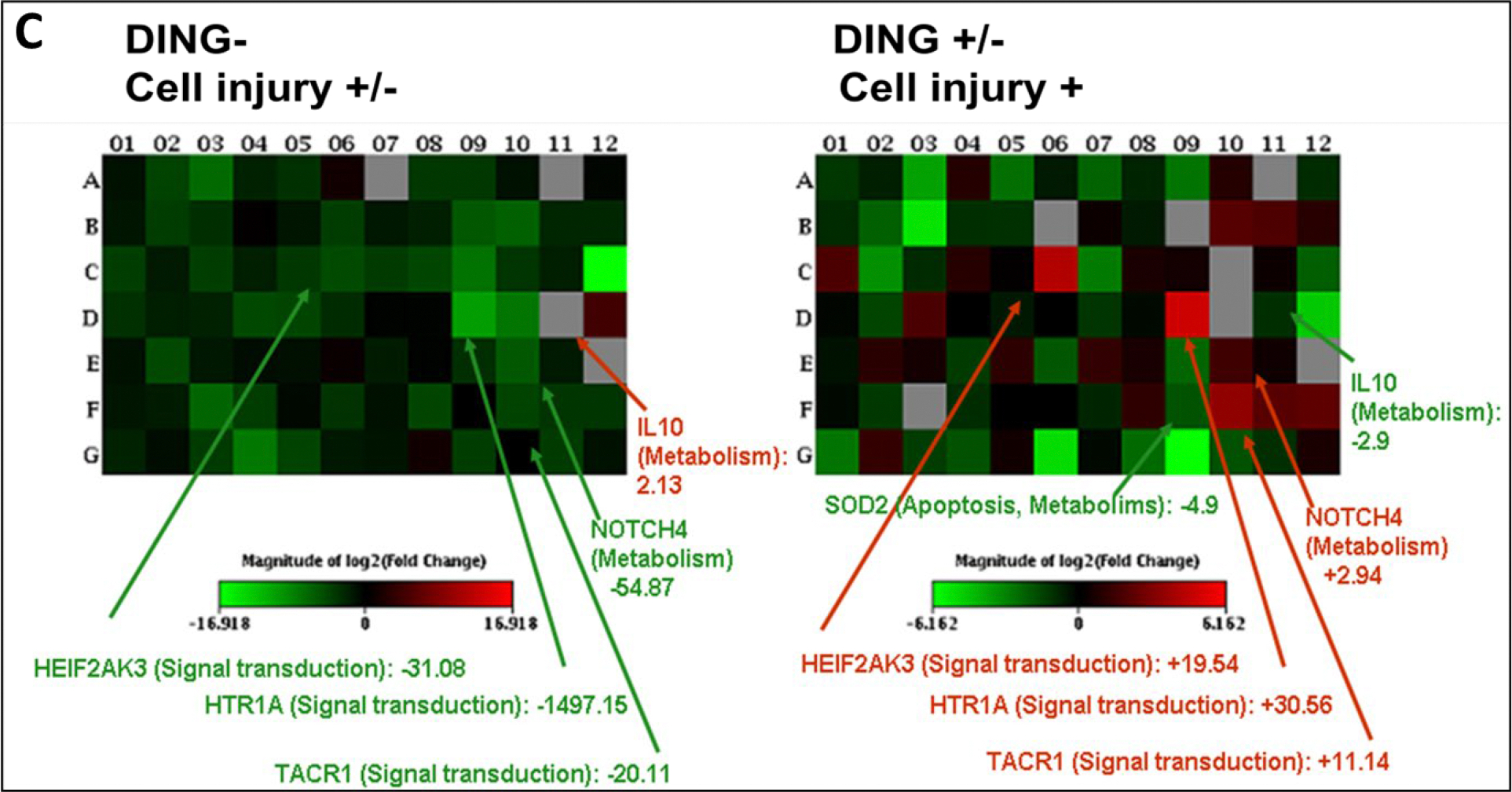
Protective effects of DING in EtOH-treated human neuronal cells in vitro. **A.** DING reverses the inhibitory effects of EtOH on cell survival. Neuronal cells were incubated with EtOH (50 mM) and/or DING (300 ng/ml). Cell viability assay in human neurons pre-incubated with DING for 24 hours and then treated with EtOH for 48 hours. An equal number of cells was plated in duplicate, and cell viability was evaluated by the Trypan blue exclusion assay. Bar 1 represents untreated cells set as 100%. Each bar represents the mean and standard deviation of three determinations in one fetus. *** p < 0.001. **B.** Impact of DING on the expression of genes involved in synaptic function in cells of human neurospheres exposed to EtOH. SYNPO, NGF, PCDH8, and GRIN2C genes are inhibited by EtOH. DING treatment reverses the suppressive effects of EtOH on synaptic genes; cells exposed to EtOH. Cells incubated with DING (3rd panels). Bar 1 represents untreated cells set as 100%. Each bar represents the mean and SE of three determinations in one fetus. * p < 0.05, ** p < 0.01, and *** p < 0.001. **C.** DING reverses the inhibitory effect of EtOH on the synaptic gene expression. Neuronal cells were transfected with pEYFP-DING, treated with EtOH for 56 h, and then RNA was analyzed for synaptogenesis pathways.

In primary cultures of both fetal neurons and neurospheres, DING by itself had little effect on cell survival but suppressed the inhibitory effects of EtOH on cell survival from 70% viable cells to 90%, compared to unexposed controls (Figure 4A). We then studied the effect of DING on the EtOH-associated changes in mRNA expression of the genes for the synaptic proteins synaptopodin (SYNPO), nerve growth factor (NGF), protocadherin 8 (PCDH8), glutamate ionotropic receptor NMDA type subunit 2C (GRIN2C), and activity-regulated-cytoskeleton-associated protein (ARC) (Figure 4B). EtOH downregulated SYNPO, NGF, PCDH8, and GRIN2C, while upregulating ARC. By itself, DING increased the expression of NGF, PCDH8, and GRIN2C, but reduced the expression of ARC. When added to EtOH, DING not only blunted the downregulation of SYNPO but also increased its expression compared to controls, while enhancing the downregulation of ARC (Figure 4B). DING also reversed the strong inhibitory effect of EtOH on expression of the serotonin receptor HTR1A, converting the effect to a 30-fold increase (Figure 4C).

### 2.5. EtOH inhibits expression of DING in fetal brain development: DING binds to the serotonin transporter.

**Figure 5:**
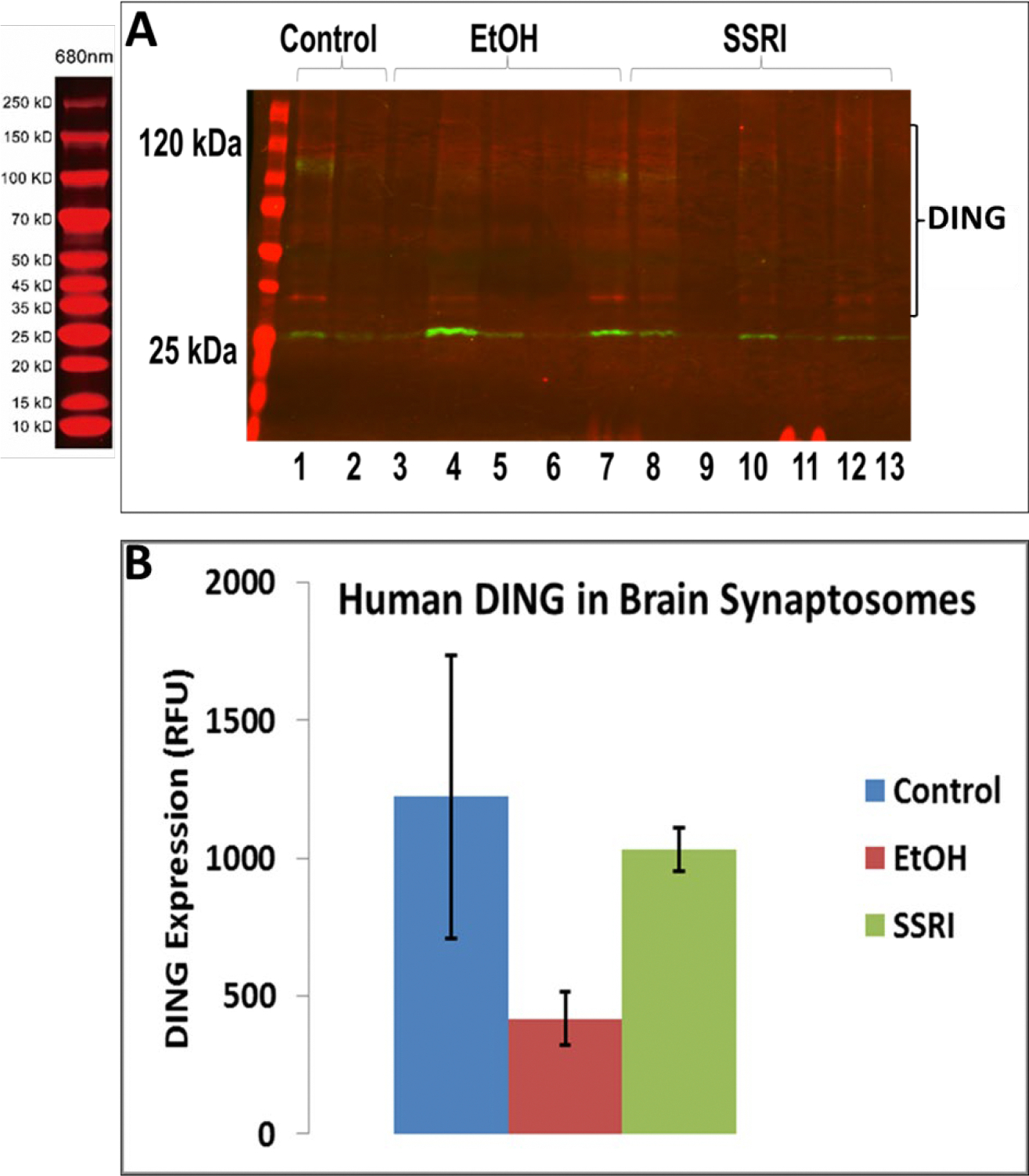

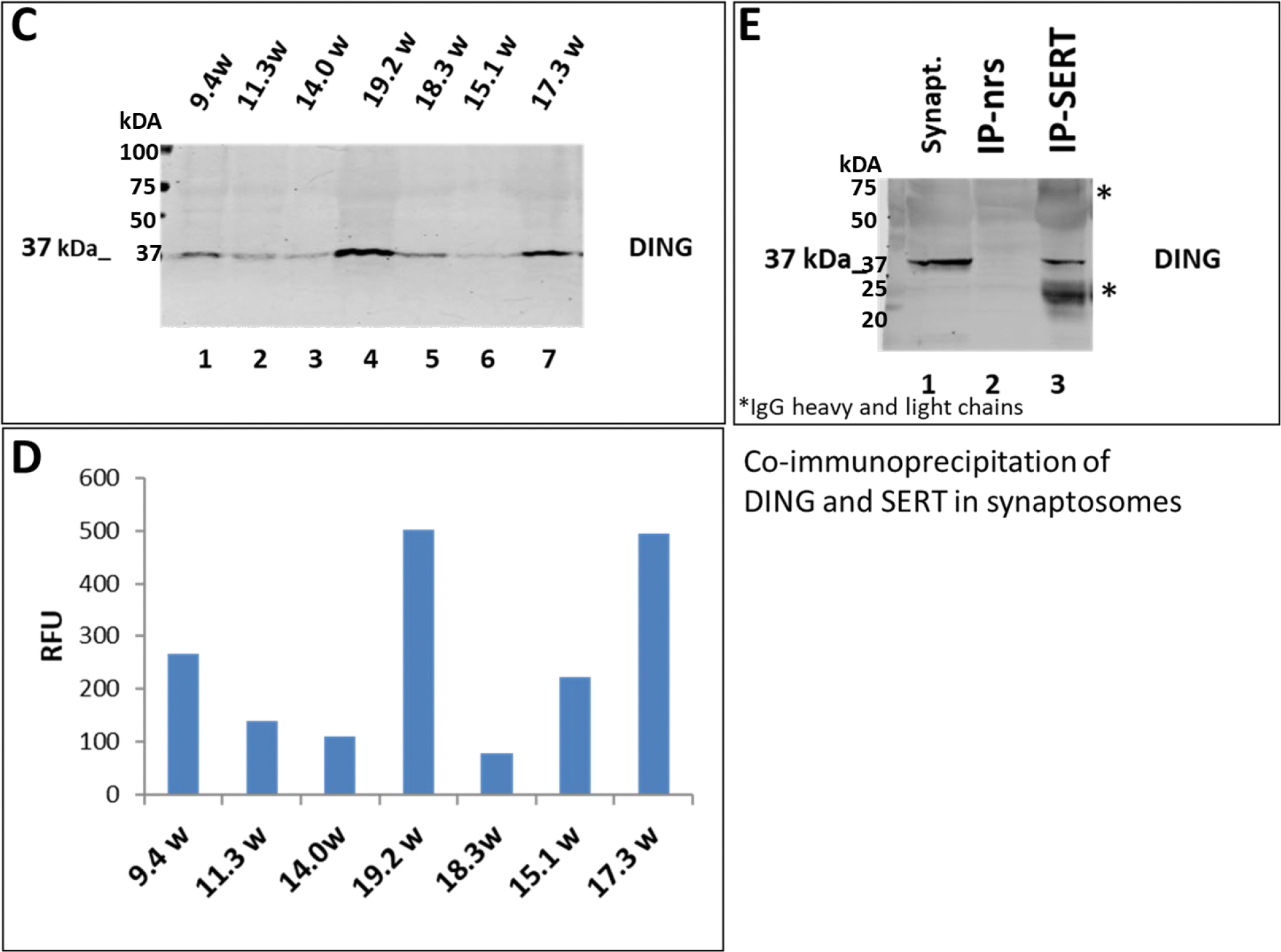
Expression of DING in synaptosomes during gestation, in the presence of EtOH or SSRI. **A.** Downregulation of DING by prenatal EtOH and SSRI exposures in fetal brain synaptosomes, assayed by qWestern blot assay. **B.** Quantification of band intensity for DING isoforms. **C.** DING expression in the human fetal brain synaptosomes with prenatal EtOH exposure from the 1^st^ and 2^nd^ trimester. Only the low molecular weight (LMW) DING isoform was expressed in synaptosomes from the developmental brain at 1st and 2nd gestation ages. Possibly, 37kD DING is a membrane-bound form of DING in both the placenta and the brain. **D.** Densitometry of band intensity shown in **C. E.** DING co-immunoprecipitated with SERT in synaptosomes. Synaptic and cytoplasmic extracts were prepared using a Syn-Per Synaptic Protein Extraction Reagent (Thermo Scientific).

DING was downregulated in prenatal EtOH- and SSRI-exposed fetal brain synaptosomes (Figure 5A). Quantifying band intensity for DING isoforms revealed a 3-fold decrease in EtOH-exposed cases and a 1.5-fold decrease in SSRI-exposed samples (Figure 5B). Interestingly, only the low molecular weight DING isoform was expressed in synaptosomes from the fetal brain at 1^st^ and 2^nd^ gestation (Figure 5C). DING was expressed at a higher level in the 2nd trimester compared to the 1st trimester (Figure 5D), and expression in the human fetal brain synaptosomes with prenatal EtOH exposure from the 1st and 2nd trimesters. Finally, in synaptosomes, DING co-immunoprecipitated with SERT (Figure 5E), suggesting a possible role for DING in controlling SERT, in addition to the serotonin receptor 5HTR1A (Figure 5C), not only in FASD but perhaps also in depression.

To understand the molecular mechanisms underlying the neuroprotective effects of DING, we looked at its biochemical features, such as its effects on MAPK signaling.

### 2.6. DING inhibits serine/threonine phosphorylation and EtOH-induced JNK2 hyperphosphorylation: A possible mechanism of neuroprotection by DING.

**Figure 6:**
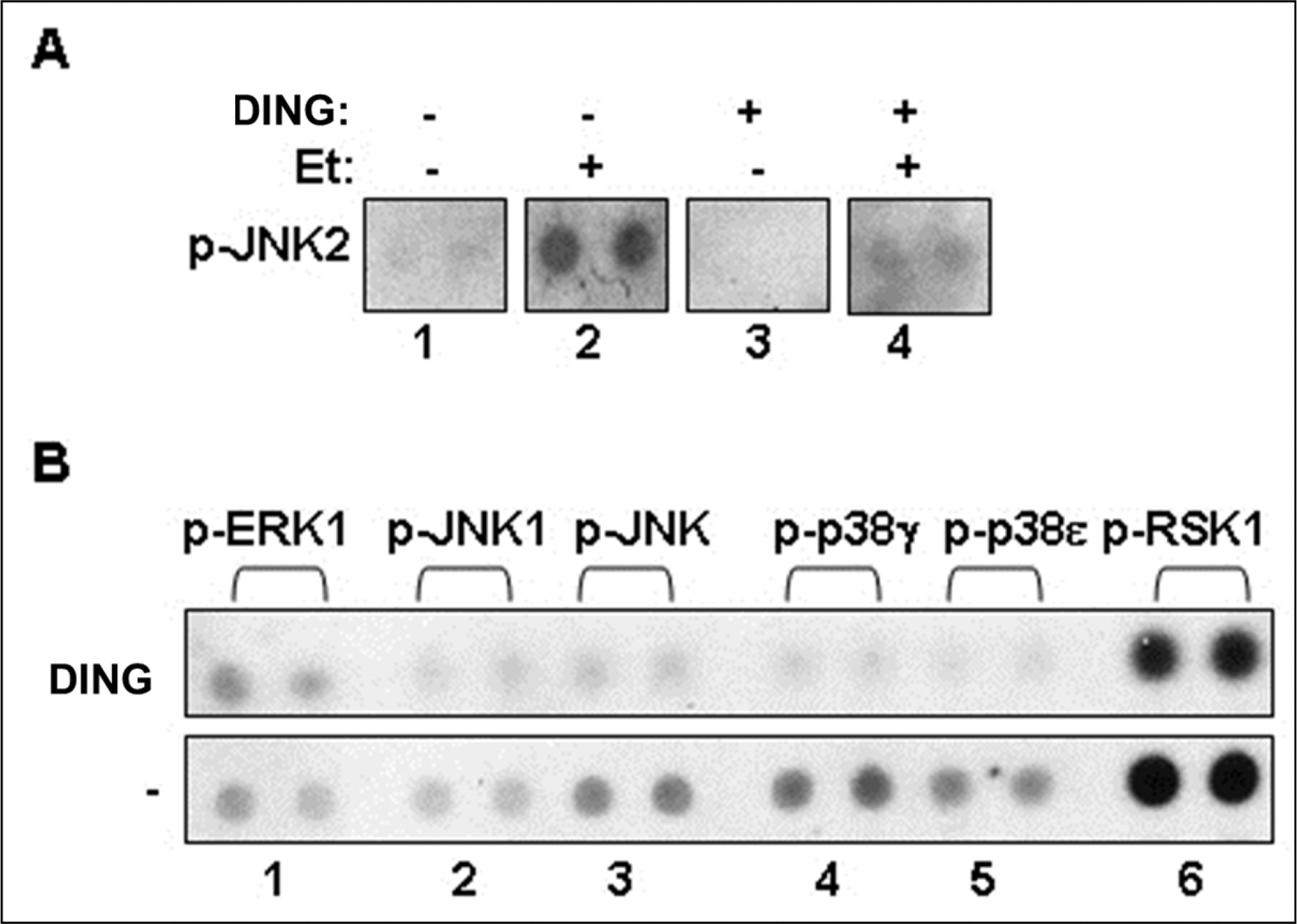
DING reverses the toxic effects of EtOH by inhibition of serine/threonine phosphorylation and EtOH-induced JNK2 hyperphosphorylation: Potential mechanism of neuroprotection of DING against EtOH-induced neuronal injury. **A.** DING-associated inhibition of JNK2 phosphorylation activated by EtOH in neuronal cells. **B** Effect of DING on serine/threonine phosphorylation of MAPK signaling in primary brain cells. Phospho-MAPK array using lysates from cells transfected with YFP or YFP-DING. DING inhibits serine/threonine phosphorylation of key MAPK signaling molecules. Thus, DING exhibits a serine/threonine-specific phosphatase activity. Phospho-MAPK array assay on ERK1, JNK1, p38, and RSK1 was performed in neuronal cells expressing DING.

JNK2 phosphorylation was increased in EtOH-exposed cells, while DING treatment inhibited JNK2 hyperphosphorylation caused by EtOH in neuronal cells (Figure 6A). DING overexpression in primary neuronal cells also inhibited basal levels of serine/threonine phosphorylation of MAPK signaling, including ERK1, JNK1, p38, and RSK1 (Figure 6B). Thus, DING was expressed and active in the developing human brain. DING expression was found in placenta, blood, and FB-Es, and in human fetal brain neurons in situ, as well as in synaptosomes and in human fetal neurons in primary cell cultures, where it was associated particularly with nuclei, mitochondria, and ER, but not in the Golgi.

## 4. Discussion

The data presented above suggest that at least one member of the DING family of proteins is present in the human fetal brain and that DING protein protects human fetal neurons *in vitro* from EtOH-induced toxicity. Thus far, there has been no evidence for multiple genes representing this family [7,12,13,15,16], and it is not yet known whether the various sizes of the DING proteins seen on western blots represent proteolytic products of a single precursor, whether different tissues express different DING proteins, nor whether the minor variations in sequences found in different species represent truly different proteins [17]. The involvement of DING in a large spectrum of diseases suggests a potential therapeutic value of this protein family and makes it important to determine the molecular mechanisms involved in its protective actions. In the present study, we have presented data on the mechanisms of neuroprotection by DING in human fetal brains *in vivo*. Early fetal exposure to EtOH was linked to increased neuronal apoptosis, which was accompanied by reduced levels of synaptic proteins and DING protein expression in the fetal brain. Our findings elucidate potential mechanisms for the neurodevelopmental injury observed in FASD and a molecular basis for the clinical observation that drugs that block DING activity are associated with an increased rate of FASD.

### Endogenous DING proteins were detected in human neurons.

Using a polyclonal antibody raised against the 27KDa DING protein from Saint John’s wart (anti-27SJ/DING; [7]) and a monoclonal antibody raised against the human DING (human phosphate binding protein; HPBP; [20,21]), DING proteins were detected in human fetal brain neurons in primary cultures. The DING proteins were present in neuronal cell bodies and processes, as determined by co-staining with the neuronal marker anti-βIII-tubulin. Within the neurons, the DING label was associated with the nucleus, the mitochondria, and the ER.

### DING protein isoforms during development.

Western blot assays revealed the presence of low and high-molecular-weight isoforms of DING (Figures 3 and 4). The most prominent and persistent band was at 37 kDa, suggesting that active DING may be a proteolytic product of a larger precursor [13].

### DING protects human neuronal cells from EtOH-induced cell injury in vitro.

In the *in vitro* primary cultures of human fetal neurons, EtOH (50 mM) produced a 50% decline in metabolic activity, while DING reversed this toxic effect of EtOH on the survival rate back to 80% of controls. Neuronal cells expressing exogenous DING, or incubated with DING, and treated with EtOH for 56 hours, were analyzed for genes involved in synaptogenesis pathways. DING prevented EtOH-induced changes in expression of synaptic genes in response to alcohol exposure. SYNPO, NGF, PCDH8, and GRIN2C expressions were inhibited by EtOH exposure, while ARC was slightly upregulated. DING reversed these effects of EtOH. This function of DING might be important in counteracting the toxic effects of EtOH on the developing brain.

### EtOH exposure suppresses DING expression in the developing human brain.

Prenatal exposure to EtOH downregulated DING mRNA expression in maternal blood, placenta, and synaptosomes, and for some DING isoforms, in the brain. These changes might provide markers for neurodevelopmental injury in the fetal brain. Only one low molecular weight DING isoform was expressed in synaptosomes, whereas low and high molecular weight DING isoforms were expressed in the human fetal brain during the 1^st^ and 2^nd^ trimesters. These were changed differentially with EtOH exposure in brain samples across GA.

### Mechanism of neuroprotection by DING phosphatase: DING inhibits serine/threonine phosphorylation and EtOH-induced JNK2 hyperphosphorylation.

To understand the molecular mechanisms underlying the neuroprotective effects of DING, we previously demonstrated that DING exhibits phosphatase activity and controls MAPK signaling in rat neurons [19]. In that study, we looked at the MAPK/ERK1/2 cascade, which modulates the expression of GSK3β, controlling neuronal progenitor proliferation and establishment of neuronal polarity during development and throughout the lifespan. It was also shown that in CNS neurons, c-Jun activity in stress-induced apoptosis is regulated by its N-terminal phosphorylation. Jun N-terminal kinases, JNKs, can regulate phosphorylation of c-Jun in CNS neurons in humans. In the present study, EtOH induced phosphorylation of JNK1 and JNK2, while DING inhibited the EtOH-induced phosphorylation of JNK2 in neuronal cells *in vitro*. Results from a phospho-MAPK array using cell lysates from cells transfected with YFP and YFP-DING indicated that DING inhibits serine/threonine phosphorylation of key MAPK signaling molecules in human neurons: ERK1, JNK1, p38, and RSK1. In the present study, pregnancy was associated with decreased serum levels of DING, which were further reduced by exposure to EtOH. These findings suggest an important role for endogenous DING in fetal protection in the setting of maternal EtOH ingestion that may be able to be augmented with exogenous DING. Thus, DING may be a promising therapeutic in the treatment/prevention of FASD.

## Conclusions

The findings in the present study indicate that exposure to EtOH reduces endogenous DING levels in the human fetus and suggest two mechanisms by which DING may protect the developing fetus from the toxic effects of EtOH exposure. First, DING reverses the negative impact of EtOH on the MAPK signaling pathway, interfering with caspase-3 cleavage, which results in apoptosis. Second, DING stabilizes the expression of synaptic genes in the brain. These findings might provide the basis for the development of DING-based therapeutic strategies, not only for the prevention of neuronal injury in FASD but also for the reduction of neuronal damage associated with alcohol toxicity later in life.

## 4. Materials and Methods

All procedures, tissues, and cell lines used in this study are summarized in Table 1.

### 4.1. Clinical recruitment.

First and second-trimester brain tissue and maternal serum were collected from women undergoing elective pregnancy termination under Temple University IRB-approved protocols (#21476: Early Gestation Alcohol Exposure: Mechanisms of Human Developmental Injury, PI Dr. Darbinian, Nune) (Table 2). A trained study coordinator conducted a face-to-face history. The amount of EtOH was calculated as the total number of drinks consumed per week multiplied by the number of weeks of exposure. A detailed questionnaire was used based on the NICHD PASS study. Each drink was estimated as the equivalent of one shot (1.5 oz of brandy or 5 oz of wine). Samples were collected between 9 and 23 weeks GA.

### 4.2. Cell Culture.

Human primary cortical neurons were prepared using a method developed by Dr. A. Darbinyan [22]. In brief, after careful removal of the meninges, intact human fetal brain tissue (embryonic age 16 weeks, approximately 13g) was incubated with Tryple Express enzyme (Invitrogen, Carlsbad, CA) and DNase I (10 U/ml; Sigma, St. Louis, MO) at 37°C for 10–20 min, followed by three washes with Hibernate E medium. Tissue trituration was performed in culture medium (Neurobasal medium containing B27 supplement and 0.25 mM Glutamax) using a glass Pasteur pipette, and cells were plated on 60 mm poly-D-lysine-coated dishes (Sigma). Cytosine arabinoside, Ara-C (Sigma) (final concentration 1 μM), was added after 16 hours for two days to reduce glial proliferation. Treatment of neuronal cultures with Ara-C (48 hours) efficiently depletes proliferating cells. At day 10 or 12 of *in vitro* culturing, more than 98% of cells were positive for neuronal marker class III β-tubulin (verified by immunocytochemical analysis. Cells were maintained in Neurobasal medium supplemented with antibiotics (10 μg/ml gentamycin, 100 units/ml penicillin, and 10 μg/ml streptomycin) and antifungal fungizone (Life Technologies, Inc.), 1 μg/ml, at 37°C in a humidified atmosphere containing 5% CO_2_.

### 4.3. Treatment of neuronal cells.

Human cortical neuronal cells in primary culture were pre-incubated with DING (300 ng/ml) for 24 hours, prior to incubation with EtOH (50 mM) for 48 h, for a total of 56 hours. The numbers of neurons and neuronal processes were measured in the presence of DING, EtOH, or both.

### 4.4. Isolation of Fetal Brain-Derived Exosomes (FB-Es) from Maternal Serum.

Human FB-Es were isolated as described previously [10].

### 4.5. Preparation of protein extracts from neuronal cells and immunoblot analysis.

Neuronal cells were incubated with EtOH for 48 hours. For preparation of whole-cell extract, cells were washed with cold phosphate-buffered saline (PBS) and solubilized in lysis buffer (50 mM Tris-HCl, pH 7.4, 150 mM NaCl, 0.1 % Nonidet P-40, and 1% protease inhibitors cocktail (Sigma). Cell debris was removed by centrifugation at 14,000 RPM for 5 min at 4°C. For western blot analysis, thirty micrograms of proteins were eluted with Laemmli sample buffer, heated at 95°C for 10 min, separated by 10% sodium dodecyl sulfate-polyacrylamide gel electrophoresis (SDS-PAGE), and transferred to supported nitrocellulose membranes (Bio-Rad) for 2 hours at 4 °C.

### 4.6. Preparation of total protein extracts from brain tissues and immunoblot analysis.

Homogenization, lysis, and western blotting were performed as previously described in our publications. The blots were subsequently washed three times, and the bound antibody was detected either with the ECL kit or with the LI-COR system. For the LI-COR system, blots were incubated with IRDye^®^ 800CW Goat Anti-Rabbit and IRDye^®^ 680RD Goat Anti-Mouse Li-COR dyes and visualized with an Odyssey^®^ CLx Imaging System (LI-COR, Inc., Lincoln, NE) using Odyssey software (LI-COR Biosciences, Lincoln, NE, USA).

### 4.7. Synaptosome extract preparation.

Human fetal synaptic vesicles and cytoplasmic extracts were prepared from snap frozen brain tissue using Syn-Per synaptic Protein Extraction kit (Reagent #87793, Thermo Scientific). Synaptic vesicle proteins (30ug) were separated by gradient SDS-PAGE (4%-20%) and transferred to nitrocellulose membrane. Proteins were detected using specific primary antibodies and secondary IRDye^®^ antibodies with the Odyssey^®^ CLx Imaging System.

### 4.8. RNA Preparation and qRT-PCR.

Total RNA was isolated using the RNeasy kit (Qiagen, Valencia, CA) with on-column DNA digestion. The RT-PCR reaction was performed with 1 μg total RNA, using One-Step FAST RT-PCR Sybr Green mix (Qiagen) on a Step One machine (Applied Biosystems). PCR conditions were activation at 95°C for 5 min, PCR 45 cycles: 95°C 10 sec, 60°C 20 sec, 72°C 30 sec, melting curve (95–65°C), cool to 40°C 30 sec. For relative quantification, the expression level of genes was normalized to the housekeeping gene β-actin. Results were presented in arbitrary units. The primers were used: β-actin, S 5′-CTACAATGAGCTGCGTGTGGC-3′, AS 5′-CAGGTCCAGACGCAGGATGGC-3′.

### 4.9. Genomic DNA isolation.

9.1. Genomic DNA was isolated using the Gentra Puregene DNA isolation kit (Qiagen Inc., Valencia, CA). Five hundred nanograms of genomic DNA were used in the PCR reactions.

### 4.10. Real-Time RT-PCR:

cDNA for q-PCR was obtained using the total RNA isolated kit (Qiagen) and 1st strand RT synthesis (Qiagen), and Synaptogenesis Pathway Kit (Qiagen) according to the manufacturer’s protocols. The expression of a focused panel of 84 human genes associated with synaptogenesis pathways was analyzed and quantified. The StepOnePlus Real-Time PCR system thermo cycler was used. GAPDH and β-actin housekeeping genes were used for normalization. Genes regulating Neurotoxicity/apoptosis (Caspase-3) levels were assessed in the brain using quantitative Real-Time RT-PCR.

### 4.11. Droplet Digital PCR (ddPCR).

For absolute quantitation of mRNA copies, ddPCR was performed using the QX200 ddPCR system. Fifty nanograms of human fetal total RNA were used with the 1st Strand cDNA Synthesis Kit (Qiagen, Valencia, CA, USA). After reverse transcription, the cDNA (300-fold dilution) aliquots were added to the BioRad master mix to conduct ddPCR (EvaGreen ddPCR Supermix, BioRad, Hercules, CA, USA). The prepared ddPCR master mix for each sample (20-μL aliquots) was used for droplet formation. PCR conditions: Activation 95 °C 5 min, PCR 45 cycles at 95 °C 10 s, 60 °C 20 s, 72 °C 30 s, melting curve (95–65 °C), cool to 40 °C 30 s. The absolute quantity of DNA per sample (copies/μL) was calculated using QuantaSoft Analysis Pro Software (AP) (Bio-Rad, Hercules, CA, USA) to analyze ddPCR data for technical errors (Poisson errors). With 20,000 droplets, the above ddPCR protocol yields a linear dynamic range of detection between 1 and 100,000 target mRNA copies/μL. The estimated error is negligible compared with other error sources, e.g., pipetting, sample processing, and biological variation. The ddPCR data were exported to Microsoft Excel (Microsoft 365) for further statistical analysis.

### 4.12. Oligonucleotides (DING full length and deletion fragments).

Oligonucleotides were prepared commercially by Oligos Etc., Inc. (Wilsonville, OR, USA).

+ strand:

5’-ATGTTTAAGCGCAACGTTCTCGCGGCATCC (*−66*)

5’-ATGGCCGATATAAACGGTGGTGGTGC (*1*)

5’-GATATAAACGGTGGTGGTGCGACACTACC (*7*)

5’-GCCTTCCTGAACAACGACTACACCAAGTTC (*118*)

5’-ACCCTGGCCGGTCTGGACGACGCGACCAA (*646*)

5’-ACCTACATGAGCCCTGATTTCGC (*616*)

− strand:

5’-TTACAGCGGACGGCCGATGCCGTTGCAGAC (*1179*)

5’-GGAGGTCAGGAACGACTGGCGTACGGGCAAG (*1119*)

5’-ATGGTTGGTGATCGCGGTGTCGTTGTTGGC (*1059*)

DING-1-N-terminal primers:

DING-1/38SJ-p27ATGup

5’ ATT ACG AAT TCC AAT ATG GCC GAT ATA AAC GGT

p38SJ-p27 ATG bot

5’ GCT CGA GAA TTC CGG CTT TTC GGT TGA TGC TGG AAC

p38SJ-p17 top

5’ ATT ACG GAA TTC GGC AAA GCC AAC ACC GCC

p38SJ-p17 bot

5’ ATC AAT TGA ATT CAG CGG ACG GCC GAT GCC GTT

p27SJ: 788 bp GI:57868105

Pseudomonas DING genes: gi:68342549, gi:229359445, gi:68344426, gi:155723471.

### 4.13. Quantitative Western Blot Assays.

Changes in apoptotic or autophagy protein levels in rat brain were measured by quantitative western blotting, as previously described [11]. The loading dose was determined by protein concentration. Proteins (30 μg) in Laemmli sample buffer were heated at 95 °C for 10 minutes, separated by gradient SDS-PAGE (4–20%), and transferred to an NC membrane. Proteins were detected using specific primary antibodies (1:1,000 dilution) and secondary IRDye^®^ dyes (IRDye^®^ 800CW Goat Anti-Rabbit and IRDye^®^ 680RD Goat Anti-Mouse Li-COR dyes, 1:10,000) with the Odyssey^®^ CLx Imaging System (LI-COR, Inc., Lincoln, NE, USA). Band intensity (normalized to Grb2) was detected, visualized, and quantified using iS Image Studio^™^ Software version 3.1.

### 4.14. Antibodies.

Monoclonal anti-human HPBP antibody for a human DING protein was a gift from Dr. Chabriere (France). A rabbit polyclonal antibody raised against our DING protein from St. John’s wort was obtained from Lampire Biological Laboratories, Inc. Pipersville, PA). Anti-HPBP human DING protein was a gift from Dr. Chabriere (Marseille, France). Anti-α-tubulin clone B512 was obtained from Sigma-Aldrich (Sigma-Aldrich Co., St. Louis, MO). Neuronal class III β-tubulin (TUJ1) monoclonal antibody (Alexa Fluor-labeled, catalog No. A488–435L) was obtained from Covance (Berkeley, CA). Mouse monoclonal anti-Grb-2 antibody was obtained from BD Biosciences/Clontech (Palo Alto, CA, USA). These primary antibodies were used in 1:1000 dilutions.

### 4.15. Neurotoxicity/apoptosis (Caspase-3) assay.

Apoptotic Caspase-3/7 levels were assessed in brain and synaptic extracts using quantitative Real-Time RT-PCR and the Caspase-Glo 3/7Assay. Genes regulating Neurotoxicity/apoptosis (Caspase-3) levels were assessed in brain and synaptic extracts using quantitative Real-Time RT-PCR and the Caspase-Glo 3/7Assay. The expression of a focused panel of 84 human genes associated with synaptic pathways (Qiagen) was analyzed and quantified by quantitative real-time RT-PCR.

### 4.16. Caspase-GLO 3/7 activity assay.

Apoptosis was assessed by analysis of activation of caspase-3 using the substrate DEVD-aminoluciferin from the Caspase-Glo^™^ 3/7 assay kit (Promega, Madison, WI, USA), according to the manufacturer’s instructions. Approximately 1,000 EtOH- or DING-1-treated cells, grown in a 24-well tissue culture plate or brain cell lysate, were analyzed in a final volume of 100 microliters of culture medium per well. One hundred microliters of Glo reagent (1:1) were added to the culture medium, and cell lysis was induced by shaking the cells for 2 min at room temperature. Subsequently, the supernatants of the different wells were transferred to the microcentrifuge tubes, and the luminescent signal was stabilized for 10 min at room temperature. Luminescence was recorded as RLU/sec on a Femtometer FB12 Luminometer (Zylux Corporation). Data were analyzed using Excel software. The histogram shows a fold increase of Caspase 3/7 activity, and the error bars show the standard deviation from three independent readings.

### 4.17. Synaptic plasticity array.

The expression of a focused panel of 84 human genes associated with synaptic pathways (Qiagen) was analyzed and quantified by quantitative real-time RT-PCR.

### 4.18. Phospho-MAPK kinase array.

Human phospho-MAPK array kit was used (R&D Systems, Inc., Minneapolis, MN, USA). Neuronal cells were lysed and used for phospho-MAPK arrays according to the manufacturer’s protocol. Neuronal cells transfected with pEYFP-C1 and YFP-DING-1 were lysed, and lysates were applied and processed over the phospho-MAPK arrays as per the manufacturer’s protocol. Quantitative representation of serine/threonine phospho-MAPK protein levels in DING-1-expressing neuronal cells was determined by densitometric analysis of each spot. The values were normalized to the levels seen in YFP-expressing cells.

### 4.19. Methylthiazolyltetrazolium (MTT) Assay.

For the MTT assay, we used a cell proliferation kit (MTT) according to the manufacturer’s protocol (Roche Applied Sciences, Indianapolis, IN USA). Neuronal cells were plated into 96-well plates in triplicate in two sets at a density of 10,000 cells/well and incubated with EtOH, at 50 mM/ml. After 48 hours, 10 μl MTT (5 mg/ml) were added to the wells (final concentration, 0.5 mg/ml) for 4 hours, and the reaction was stopped by the addition of 100 μl of solubilization solution. Viable cells with active mitochondria cleave the tetrazolium ring into a visible dark blue formazan reaction product, which was quantified by spectrophotometry in a Dynex MRX Revelation microplate reader (Dynex Technologies, Chantilly, VA) at 570 nm with a reference wavelength of 650 nm. The relative cell viability (percent) was determined as the ratio of the average absorbance for treated cells to that for mock, untreated cells.

### 4.20. Cell viability assay (Trypan blue exclusion viability assay).

The trypan blue exclusion assay will be performed to assess cell viability. This viability assay measures the percentage of a cell suspension that is able to exclude Trypan blue dye. In brief, the cell suspension will be diluted 1:1 with 0.4% Trypan blue, and cells will be counted with a hemocytometer. The averages and standard deviations are shown.

### 4.21. Neuronal cell morphology analysis.

Neuronal cell complexity and outgrowth were evaluated after three days of transfection or treatment. The number of neurites in neuronal cells was determined by confocal microscopy, and the values were analyzed by ImageJ software (NIH). Experiments were repeated two times. Effects of EtOH and DING-1 overexpression on the neurite outgrowth in neurons compared with the control were studied in 50 neurons per group. The average neurite length is shown.

### 4.22. Immunocytochemistry.

Immunocytochemistry was performed using the avidin-biotin-peroxidase complex system according to the manufacturer’s instructions (Vectastain Elite ABC Peroxidase Kit, Vector Laboratories Inc, Burlingame, Calif). Fluorescence detection was performed as described previously [7]. Cells were seeded in poly-L-lysine-coated glass slide chambers. After 24 h incubation, cells were fixed and washed in PBS. Fluorescence images were captured using an inverted fluorescent Nikon microscope with deconvolution software (SlideBook 4.0.1.34; Intelligent Imaging, Denver, CO, USA). Fluorescence images of cells were visualized with an inverted Olympus fluorescence microscope using IPLAB software. Contrast and brightness were adjusted equally for all images using Adobe Photoshop version 5.5.

### 4.23. Statistical Analysis.

Statistical analysis was performed using SPSS Statistics from IBM Corp., released in 2017 for Windows, Version 25.0 (Armonk, NY, USA). All data are represented as the mean ± SD for all performed repetitions. Means were analyzed by a one-way ANOVA, with Bonferroni correction, where appropriate. Statistical significance was defined as *p* < 0.05. Sample numbers are indicated in the figure legends.

### 4.24. Ethics: Human Subjects.

Consenting mothers were enrolled at between 9 and 23 weeks’ gestation, under a protocol approved by our Institutional Review Board (IRB). This protocol involved no invasive procedures other than routine care. Maternal EtOH exposure was determined with a face-to-face questionnaire that also included questions regarding many types of drugs/medications used [18]. The questionnaire was adapted from that designed to identify and quantify maternal EtOH exposure in the NIH/NIAAA Prenatal Alcohol and SIDS and Stillbirth (PASS) study [23].

All procedures involving the collection and processing of blood and brain tissues were done according to NIH Guidelines through a trained Study Coordinator. All investigators were trained annually to complete Citi Program - Human Subject training, Biohazard Waste Safety Training and Bloodborne Pathogens Training, and all other required training. Written informed consent was obtained from the patients for the studies, and de-identified samples were used for this publication.

#### Eligibility Criteria.

All subjects were pregnant. Subjects were excluded if they had an active urinary tract infection in history, nitrates or WBCs on clinical UA; no prisoners; no adults who were cognitively impaired or physically unable to provide consent to participate; no patients with severe blood disorders (e.g., hemophilia). The blood and placenta samples were obtained according to NIH Guidelines by a trained Study Coordinator. Samples were collected regardless of sex, ethnicity, and race.

#### Treatment Plan.

Each patient was asked to sign a separate consent form for research on blood and tissue samples. Blood obtained was processed for the collection of serum. No invasive procedures were performed on the mother, other than those used in her routine medical care. Placenta tissues were processed for protein isolation.

#### Risk and Benefits.

There was a small risk of loss of privacy, as with any research study with protected health information. Samples were deidentified before they were sent to the lab for analysis. There were no additional risks of blood sampling as this was only performed in subjects with clinically indicated venous access. There was a small risk from obtaining 2–3 cc of blood by a well-trained Study Coordinator, who collected all samples.

There was no direct benefit to the research subjects from participation, but there is potential benefit for the future FASD subjects and the general population. This research represents a reasonable opportunity to further the understanding, prevention, or alleviation of a serious problem affecting the health or welfare of FASD patients.

#### Informed Consent.

Consent forms were maintained by the Study Coordinator in a locked space and were not sent to the investigators with the samples.

## Figures and Tables

**Table 1:** Tissues and cell lines used in the study.

Tissues	Cell lines, cell cultures	Samples	Assays
Human maternal serum	Human cortical neurons	Human fetal brain-derived exosomes	Synaptic plasticity array
Human fetal brain	Human neurospheres	Human Synaptosomes	Neurotoxicity array
Human placenta		RNA	Caspase-3/7 assay
Human fetal-brain exosomes		Genomic DNA	Cell viability assay
Human synaptosomes		Protein lysate	MTT assay
			ddPCR
			qRT-PCR
			qWestern blot assay
			Immunocytochemistry

**Table 2:** Subjects used in the EtOH and SSRI experiments: clinical characteristics.

A		
Subjects: Clinical characteristics	Cases: EtOH-consumers (n=20)	Controls: no EtOH (n=20)
Maternal Age (years ±SD)	25.43 ± 1.15	23.37 ± 2.41
Gestational Age (weeks ±SD)	15.33 ± 1.12	15.85 ± 1.57
Race: White vs Black (%)	50/50	50/50
Fetal Sex (male/female, %)	50/50	50/50
B		
	Drug/Medication	Subjects (number)
Control	-	6
SSRI	Celexa, Lexapro, Sertraline	6

**Maternal blood, fetal brain, and placenta tissues were used in studies. A.** Maternal blood samples from EtOH-exposed cases (n=20) and Controls (n=20) were matched individually for fetal sex, GA, and maternal age. PCR for the SRY gene on the Y chromosome for sex determination was performed for EtOH cases and control samples. Subjects were either Caucasian or African American. Other races were not included. **B.** SSRI exposure groups for most experiments using qWestern blot assays for brain, placenta, and blood tissues (n=6 total) vs. Controls (n=6).

## Data Availability

This study collected demographic, behavioral, and laboratory data from normal, healthy women and from women who drank alcohol during pregnancy. Our research team has developed a data-sharing plan. We recognize that additional benefits from data sharing may arise in the future that are not apparent at this time, and are prepared to work specifically with NIH in addressing all requests for raw data. At the present time, we have not deposited the raw data in an existing databank but will make the data available to other investigators on request, in a manner consistent with NIH guidelines. Consistent with NIH policy, shared data will be rendered “free of identifiers that would permit linkages to individual research participants and variables that could lead to deductive disclosure of the identity of individual subjects” Intellectual property and data generated under this project will be administered in accordance with both University and NIH policies, including the NIH Data Sharing Policy and Implementation Guidance of 5 March 2003, and 0925–0001 and 0925–0002 (Rev 07/2022 through 01/31/2026). With this caveat observed, data will be made available to the NIH/NICHD/NIAAA. Sufficient identifiers will be provided to the NIH so that research participants can be assigned a Global Unique Identifier (GUID), which is a universal subject ID that protects personally identifiable information (PII). Using the GUID, NDAR can bring together multiple types of data collected from a single participant, regardless of where and when those data were collected. Biological samples (blood, serum, exosomes, and RNAs) and data that are shared will be completely free of identifiers that would permit linkages to individual research participants. We will make biological samples, deidentified data, and associated documentation available to users only under a data-sharing agreement that provides for (1) a commitment to using the data only for research purposes, (2) a commitment to securing the data using appropriate computer technology; and (3) a commitment to destroying or returning remaining samples after analyses are completed. Intellectual property and data generated under this project will be administered in accordance with both University and NIH policies, including the NIH Data Sharing Policy and Implementation Guidance of 5 March 2003. As the FAIR data bank receives approval from the NIH, the data will be made available to that group as well. The NIH implemented a new policy for Data Management and Sharing, effective on January 25, 2023 (https://grants.nih.gov/grants/guide/notice-files/NOT-OD-21-014.html). We will adopt that policy also. Data will be also available at https://www.mdpi.com/ethics accessed on 1 January 2025.

## Abbreviations:

ddPCR: digital droplet polymerase chain reaction
DING: protein containing the highly conserved motif Asp-Ile-Asn-Gly-Gly-Gly, p38SJ protein; p38hu
ELISA: enzyme-linked immunosorbent assay
EtOH: ethanol, alcohol
FAS: fetal alcohol syndrome
FASD: fetal alcohol spectrum disorder
FB-Es: fetal brain exosomes
GA: gestation age
5-HT: serotonin (5-HT) receptor
HTR1A: 5-hydroxytryptamine serotonin receptor 1A
mtDNA: mitochondrial DNA
MTT: Methylthiazolyltetrazolium assay
NC: nitrocellulose membranes
NGF: nerve growth factor
OGG1: 8-oxoguanine DNA glycosylase-1
PAGE: polyacrylamide gel electrophoresis
PAE: prenatal alcohol exposure
qRT-PCR: quantitative reverse transcription polymerase chain reaction
RFU: relative fluorescence units
SERT: serotonin transporter
SSRI: Selective Serotonin Reuptake Inhibitor
